# TGFβ Signaling Dysregulation May Contribute to COL4A1-Related Glaucomatous Optic Nerve Damage

**DOI:** 10.1167/iovs.65.5.15

**Published:** 2024-05-08

**Authors:** Mao Mao, Yien-Ming Kuo, Alfred K. Yu, Cassandre Labelle-Dumais, Yvonne Ou, Douglas B. Gould

**Affiliations:** 1 Department of Ophthalmology, University of California, San Francisco, San Francisco, California, United States; 2 Department of Anatomy, Institute for Human Genetics, Cardiovascular Research Institute, and Bakar Aging Research Institute, University of California, San Francisco, San Francisco, California, United States

**Keywords:** Gould syndrome, basement membrane, COL4A1, COL4A2, TGFβ, TGFBR2, glaucoma

## Abstract

**Purpose:**

Mutations in the genes encoding type IV collagen alpha 1 (COL4A1) and alpha 2 (COL4A2) cause a multisystem disorder that includes ocular anterior segment dysgenesis (ASD) and glaucoma. We previously showed that transforming growth factor beta (TGFβ) signaling was elevated in developing anterior segments from *Col4a1* mutant mice and that reducing TGFβ signaling ameliorated ASD, supporting a role for the TGFβ pathway in disease pathogenesis. Here, we tested whether altered TGFβ signaling also contributes to glaucoma-related phenotypes in *Col4a1* mutant mice.

**Methods:**

To test the role of TGFβ signaling in glaucoma-relevant phenotypes, we genetically reduced TGFβ signaling using mice with mutated *Tgfbr2*, which encodes the common receptor for all TGFβ ligands in *Col4a1^+/G1344D^* mice. We performed slit-lamp biomicroscopy and optical coherence tomography for qualitative and quantitative analyses of anterior and posterior ocular segments, histological analyses of ocular tissues and optic nerves, and intraocular pressure assessments using rebound tonometry.

**Results:**

*Col4a1^+/G1344D^* mice showed defects of the ocular drainage structures, including iridocorneal adhesions, and phenotypes consistent with glaucomatous neurodegeneration, including thinning of the nerve fiber layer, retinal ganglion cell loss, optic nerve head excavation, and optic nerve degeneration. We found that reducing TGFβ receptor 2 (TGFBR2) was protective for ASD, ameliorated ocular drainage structure defects, and protected against glaucomatous neurodegeneration in *Col4a1^+/G1344D^* mice.

**Conclusions:**

Our results suggest that elevated TGFβ signaling contributes to glaucomatous neurodegeneration in *Col4a1* mutant mice.

Glaucoma is a leading cause of blindness and affects approximately 80 million people worldwide.^1^ Glaucoma can be broadly classified as primary or secondary glaucoma, the latter including other conditions such as anterior segment dysgenesis (ASD). ASD is a spectrum of developmental disorders affecting ocular structures anterior to the vitreous. Malformation of the ocular anterior segment may impact vision due to disruption of the visual axis. In addition, malformation or blockage of the ocular drainage structures may disrupt aqueous humor outflow, resulting in high intraocular pressure (IOP) and glaucoma that may lead to irreversible blindness.^2^^–^^5^ Approximately 50% of patients with ASD develop severe early-onset glaucoma that is refractory to treatment.^6^^,^^7^ Importantly, cellular pathways that are acutely dysregulated in developmental glaucoma may also be chronically dysregulated in primary open-angle glaucoma.^8^^,^^9^ Thus, understanding the biological processes underlying ASD and developmental glaucoma may help develop effective treatments for age-related primary open-angle glaucoma. Although the detailed mechanisms remain elusive, multiple factors involved in ASD have been reported, including the type IV collagens and other extracellular matrix (ECM)-related proteins.^5^^,^^10^^–^^12^

Type IV collagens are fundamental components of basement membranes (BMs)—specialized sheets of ECM that provide structural support to surrounding cells and regulate cellular behaviors.^13^ The collagen type IV alpha chains are encoded by six genes (*COL4A1* to *COL4A6*) in mammals.^14^ COL4A1 and COL4A2 are present in almost all BMs throughout the body, whereas other type IV collagen alpha chains have more restricted expression.^14^ One COL4A2 and two COL4A1 molecules assemble into collagen α1α1α2(IV) heterotrimers in the endoplasmic reticulum (ER) before being secreted into the extracellular space where they form an intricate network and incorporate into the BM.^15^^,^^16^ By interacting with other BM components, cell surface receptors, or morphogens, type IV collagens can regulate multiple biological processes such as axon pathfinding, cell differentiation, and tissue morphogenesis.^17^^–^^19^

Consistent with their ubiquitous expression pattern, mutations in *COL4A1* and *COL4A2* cause a clinically heterogenous multisystem disorder characterized by cerebrovascular, ocular, renal, and muscular manifestations^20^^–^^24^ that are collectively referred to as Gould syndrome.^25^^,^^26^ After cerebrovascular defects, ocular abnormalities are the most frequent clinical findings in individuals with Gould syndrome.^22^^,^^23^ Ocular features are highly variable and include microphthalmia, ASD, cataracts, strabismus, myopia, retinal artery tortuosity, retinal detachments, optic coloboma, optic nerve hypoplasia, and glaucoma. These features can differ even between individuals from the same family.^27^^–^^30^ Most *COL4A1* or *COL4A2* mutations affect protein folding and impair collagen α1α1α2(IV) secretion, which might trigger cytotoxicity in some cell types,^31^^,^^32^ and extracellular deficiency, which can alter BM physical properties and/or functions. Although the pathogenic mechanisms contributing to Gould syndrome are largely unknown, we recently reported that elevated transforming growth factor beta (TGFβ) signaling contributes to ocular and central nervous system vascular pathologies in *Col4a1* mutant mice.^33^^–^^35^

TGFβ superfamily ligands regulate a variety of biological activities in development and in pathological conditions. We recently showed that TGFβ signaling is elevated in both the brain and developing anterior segments of *Col4a1* mutant mice.^33^^–^^35^ Reducing TGFβ signaling genetically (using heterozygous *Tgfb1* or *Tgfb2* null mice) or pharmacologically (using a pan-TGFβ neutralizing antibody) partially ameliorated ASD and cerebrovascular manifestations in *Col4a1* mutant mice.^33^^–^^35^ Notably, we showed distinct contributions of TGFβ1 and TGFβ2 to the ASD phenotypes in *Col4a1* mutant mice, suggesting that they have different roles in ocular development, which might explain the partial protective effect seen in each model.^33^ To determine whether elevated TGFβ signaling also contributes to elevated IOP and glaucoma associated with Gould syndrome,^23^^,^^24^^,^^29^ here we tested the effect of genetically reducing TGFβ receptor 2 (TGFBR2), the common receptor for all three TGFβ ligands,^36^ on glaucoma-relevant phenotypes in *Col4a1* mutant mice.

## Methods

### Animals

All experiments were conducted in compliance with the ARVO Statement for the Use of Animals in Ophthalmic and Vision Research and approved by the Institutional Animal Care and Use Committee at the University of California, San Francisco (protocols AN159737 and AN182181). All lines were backcrossed on the C57BL/6J (B6) background for at least five generations. The *Col4a1^+/G1344D^* mutant mouse strain has been described previously.^37^^,^^38^ Mice carrying a conditional allele of the *Tgfbr2* gene (*Tgfbr2^flox^*)^39^ with *LoxP* sites flanking exons 2/3 (exon 2 in the short transcript and exon 3 in the long transcript) were bred to the ubiquitous *Actb^Cre^* line^40^ and *Col4a1^+/G1344D^* mice to generate *Col4a1^+/+^* and *Col4a1^+/G1344D^* mice with no or one copy of the mutant *Tgfbr2* allele. All animals were maintained in full-barrier facilities free of pathogens on a 12-hour light/dark cycle with ad libitum access to food and water. Both male and female mice were used for all experiments.

### Slit-Lamp Biomicroscopy

Ocular anterior segment examinations were performed on mice 1.3 to 1.5 months old using a slit-lamp biomicroscope (Topcon SL-D7; Topcon Medical Systems, Oakland, NJ, USA) attached to a digital SLR camera (Nikon D200; Nikon, Melville, NY, USA). ASD severity was subjectively determined based on the level of iris vessel dilation and tortuosity, pupil dilation, lens opacity, and anterior chamber enlargement, as previously described.^25^^,^^33^

### Ocular Biometry by Optical Coherence Tomography

Ocular biometry was performed using Envisu R4300 spectral-domain optical coherence tomography (SD-OCT; Leica Microsystems, Research Triangle Park, NC, USA). Mice were anesthetized using ketamine–xylazine (100 mg/kg and 50 mg/kg, respectively) and their pupils were dilated with 1% tropicamide. Central corneal thickness, ocular axial length, anterior chamber depth, lens thickness, and vitreous chamber depth were measured as previously described.^33^^,^^41^ The thickness of retinal layers was measured as described previously.^42^ Briefly, optic nerves were centered in both vertical and horizontal axes. Radial volume scans were performed to capture images along the nasal–temporal and superior–inferior axes of the retina. Measurements were taken at 0.25 mm and 0.50 mm from the optic nerve head in nasal, temporal, superior, and inferior retina and averaged for each eye. We assessed the thickness of the total retina (from the inner limiting membrane to the Bruch's membrane), ganglion cell complex (GCC) layer (consisting of the nerve fiber layer [NFL], retinal ganglion cell layer, and inner plexiform layer), and outer nuclear layer.

### IOP Measurements

Mice were anesthetized with a steady flow of 2% isoflurane in oxygen, and IOP was measured using a rebound tonometer (iCare TONOLAB; Colonial Medical Supply, Franconia, NH, USA) within 5 minutes of isoflurane exposure. All measurements were taken during morning sessions. The IOP value for each eye was averaged from three measurements, each averaged from six consecutive readings.

### Histological Analyses

Eyes were enucleated at the indicated ages. The eyes were fixed in half-strength Karnovsky fixative (2% paraformaldehyde and 2.5% glutaraldehyde) in 0.1-M phosphate buffer (pH 7.4) for 24 to 48 hours at room temperature and stored at 4°C. The eyes were dehydrated in a graded series of ethanol and embedded in Technovit 7100 Methacrylate (Kulzer Technik, Hanau, Germany). Then, 2-µm sections were collected from the level of the optic nerve head and stained with hematoxylin and eosin (H&E). Five to eight consecutive sections per eye were evaluated for ocular pathology. For iridocorneal adhesion length, both iridocorneal angles from the same eye were measured. For retinal NFL thickness measurement, we used the InteredgeDistance 1.2 macro for ImageJ (National Institutes of Health, Bethesda, MD, USA) according to instructions.

Cross-sections of optic nerves were examined for glaucomatous damage as previously described.^43^ Briefly, the majority of the brain was removed, leaving a thin layer of tissue covering the optic nerves. The remaining tissues were fixed in half-strength Karnovsky fixative overnight, and the optic nerves were dissected and kept in fixative for at least 24 hours at 4°C. Optic nerves were post-fixed in osmium tetroxide, dehydrated with a graded series of ethanol, and embedded in Embed 812 resin (Electron Microscopy Sciences, Hatfield, PA, USA). Then, 1-µm sections were stained with 1% paraphenylenediamine (PPD). We calculated the area of each optic nerve and counted the number of healthy axons in 10% area of each nerve using 18 non-overlapping, evenly distributed images.

### Western Blot Analyses

Western blot analyses for P7 anterior segments were performed as described previously with some modifications.^33^ Briefly, P7 anterior segments were dissected and lysed in radioimmunoprecipitation assay buffer (Sigma-Aldrich, St. Louis, MO, USA) supplemented with Halt Protease and Phosphatase Inhibitor Cocktail (Thermo Fisher Scientific, Waltham, MA, USA), EDTA, and 2-mM phenylmethylsulfonyl fluoride. Then, 6 µg of total proteins were separated on Bolt Bis-Tris Plus Protein Gels, 4-12% (Thermo Fisher Scientific), under reducing conditions and transferred onto polyvinylidene difluoride membranes (Bio-Rad, Hercules, CA, USA). Membranes were blocked in 10% BSA in Tris-buffered saline with 0.1% Tween 20 (TBST) overnight at 4°C and incubated with rabbit anti-TGFBR2 antibody (AF532-SP, 1:100; R & D Systems, Minneapolis, MN, USA) in 5% BSA in TBST at 4°C for 48 hours. After washes in TBST, membranes were incubated in horseradish peroxidase–conjugated secondary antibodies (bovine anti-goat, NC960914, 1:10000; Jackson ImmunoResearch Laboratories, West Grove, PA, USA) for 1 hour at room temperature. Immunoactivity was visualized by chemiluminescence (MilliporeSigma Luminata Forte; Thermo Fisher Scientific). An antibody against glyceraldehyde 3-phosphate dehydrogenase (GAPDH; MAB374, 1:100,000; Sigma-Aldrich) and corresponding secondary antibody (715-035-150, 1:10,000; Jackson ImmunoResearch Laboratories) were used as loading controls. Densitometric analyses were performed on low-exposure images using Bio-Rad Quantity One analysis software.

### RNA Extraction and Quantitative PCR

Total RNA was extracted using the RNeasy Plus Micro Kit (Qiagen, Hilden, Germany) and reverse transcribed using the Bio-Rad iScript cDNA Synthesis Kit. Quantitative PCR (qPCR) was performed on a Bio-Rad CFX96 Real-Time Detection System using Bio-Rad SsoFast EvaGreen Supermix as described previously.^33^ Primers used are listed in Supplementary Table S1. *Gapdh* was used as a housekeeping gene.

### Statistical Analyses

Statistical analyses were performed using Prism 8.0 (GraphPad, Boston, MA, USA). Statistical differences between two groups were determined using the two-tailed unpaired Student's *t*-test or Mann–Whitney test. Multiple-group comparisons were performed using one way ANOVA with Sidak's multiple comparison post hoc test, and Kruskal–Wallis test with Dunn's multiple comparison post hoc test for parametric and non-parametric data, respectively. Fisher's exact tests were performed for categorical data. Data are presented as mean ± SD, and *P* < 0.05 was considered statistically significant.

## Results

### Genetically Reducing *Tgfbr2* Partially Rescues Anterior Segment Defects in *Col4a1^+/G1344D^* Mice

**Figure 1.**
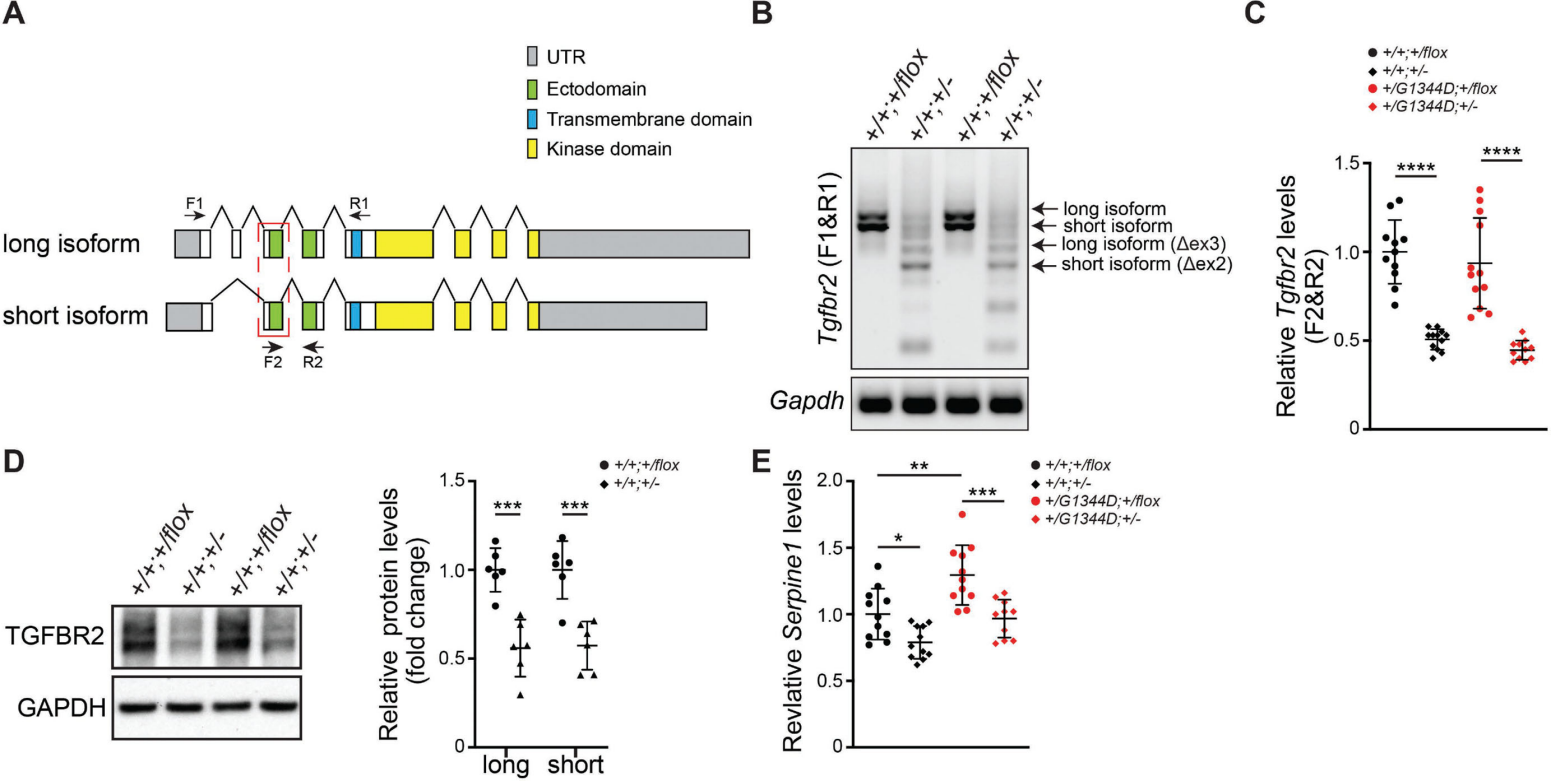
Validation of Cre-mediated recombination of the *Tgfbr2^flox^* allele. (**A**) Genomic structures of *Tgfbr2* isoforms with the recombined exon highlighted in the *red dashed box*. *Arrows* indicate primer pairs used in **B** (F1 and R1) or **C** (F2 and R2). (**B**) Reverse transcription PCR analysis using primers located in common exons (F1 and R1) flanking the floxed exon resulted in amplification of a 553-bp fragment and a 478-bp fragment in the absence of Cre, showing expression of both the long and short isoforms in P7 anterior segments. In the presence of Cre, the floxed exon was excised, resulting in two additional PCR products with smaller sizes (384 bp and 309 bp for the long and short isoforms, respectively); *n* = 5 *Col4a1*^+/+^*;Tgfbr2^+/flox^* samples and *n* = 7 *Col4a1*^+/+^*;Tgfbr2^+/^^−^* samples. (**C**) qPCR analysis using a forward primer located in the floxed exon confirmed successful recombination in anterior segments from P7 *Col4a1^+/+^* and *Col4a1^+/G1344D^* mice. Only wild-type alleles can be amplified using these primers (F2 and R2), and the amount of wild-type mRNA was reduced to ∼50% in mice with Cre expression compared to those without Cre; *n* = 11 or 12 per genotype. (**D**) Representative images and quantification of Western blots using an antibody recognizing the extracellular domain of TGFBR2 showing reduced TGFBR2 protein levels in the presence of CRE; *n* = 6 per genotype. (**E**) qPCR analysis of a major TGFβ target gene, *Serpine1*, in P7 anterior segments from *Col4a1* mice with or without inactivated *Tgfbr2*. *Serpine1* mRNA levels were increased in *Col4a1^+/G1344D^;Tgfbr2^+/flox^* mice compared to controls (*Col4a1^+/+^;Tgfbr2^+/flox^* mice)*.* The increase was prevented by *Tgfbr2* inactivation (comparing *Col4a1^+/G1344D^;Tgfbr2^+/flox^* to *Col4a1^+/G1344D^;Tgfbr2^+/^^−^* mice). Two replicates per genotype group are shown in **B** and **D**. Data are shown as fold expression relative to *Col4a1^+/+^;Tgfbr2^+/flox^* and are presented as mean ± SD; *n* = 10 to 12 per genotype. *P < 0.05; **P < 0.01; ***P < 0.001; ****P < 0.0001, one-way ANOVA with Sidak's multiple comparison test.

TGFβ signaling is initiated when TGFβ ligands bind to tetrameric cell surface receptors formed by two type II and two type I receptors. Ligand-bound type II receptors activate type I receptors that recruit and activate downstream signaling mediators and regulate target gene expression.^44^ We sought to decrease TGFβ signaling by deleting the *Tgfbr2* gene that encodes the type II receptor using *Tgfbr2^flox^* mice^39^ and ubiquitously expressed Cre recombinase (*Actb^Cre^*)^40^ in mice carrying the *Col4a1^G1344D^* mutation.^38^ For simplicity, hereafter the *Actb^Cre^;Tgfbr2^+/flox^* progeny are referred to as *Tgfbr2^+/^^−^* and *Actb^−^;Tgfbr2^+/flox^* as *Tgfbr2^+/flox^*. *Col4a1^+/G1344D^* mice have reduced viability, which was not significantly affected by *Tgfbr2* heterozygosity (Supplementary Fig. S1). *Tgfbr2* encodes two differentially spliced transcripts that are both expressed in ocular anterior segments at postnatal day 7 (P7) (Fig. 1A). Cre-mediated recombination deletes exons 2/3, which encode the majority of the ectodomain for both isoforms (Fig. 1B). Similarly, qPCR analysis showed that wild-type *Tgfbr2* mRNA levels were reduced to 50% when Cre was present, suggesting that recombination is efficient and that there is no compensatory upregulation of the non-targeted allele (Fig. 1C). Consistently, western blot analysis using an antibody recognizing the TGFBR2 extracellular domain showed that protein levels were reduced to approximately 50% in the presence of Cre (Fig. 1D). Furthermore, consistent with our previous findings,^33^^,^^34^ expression of *Serpine1*, a major TGFβ target gene, was elevated in anterior segments from *Col4a1^+/G1344D^* mice compared to *Col4a1^+/+^* mice, and we showed that *Tgfbr2* heterozygosity reduced *Serpine1* levels (Fig. 1E). Together, these data demonstrate successful *Tgfbr2* deletion and reduction of TGFβ signaling.

**Figure 2.**
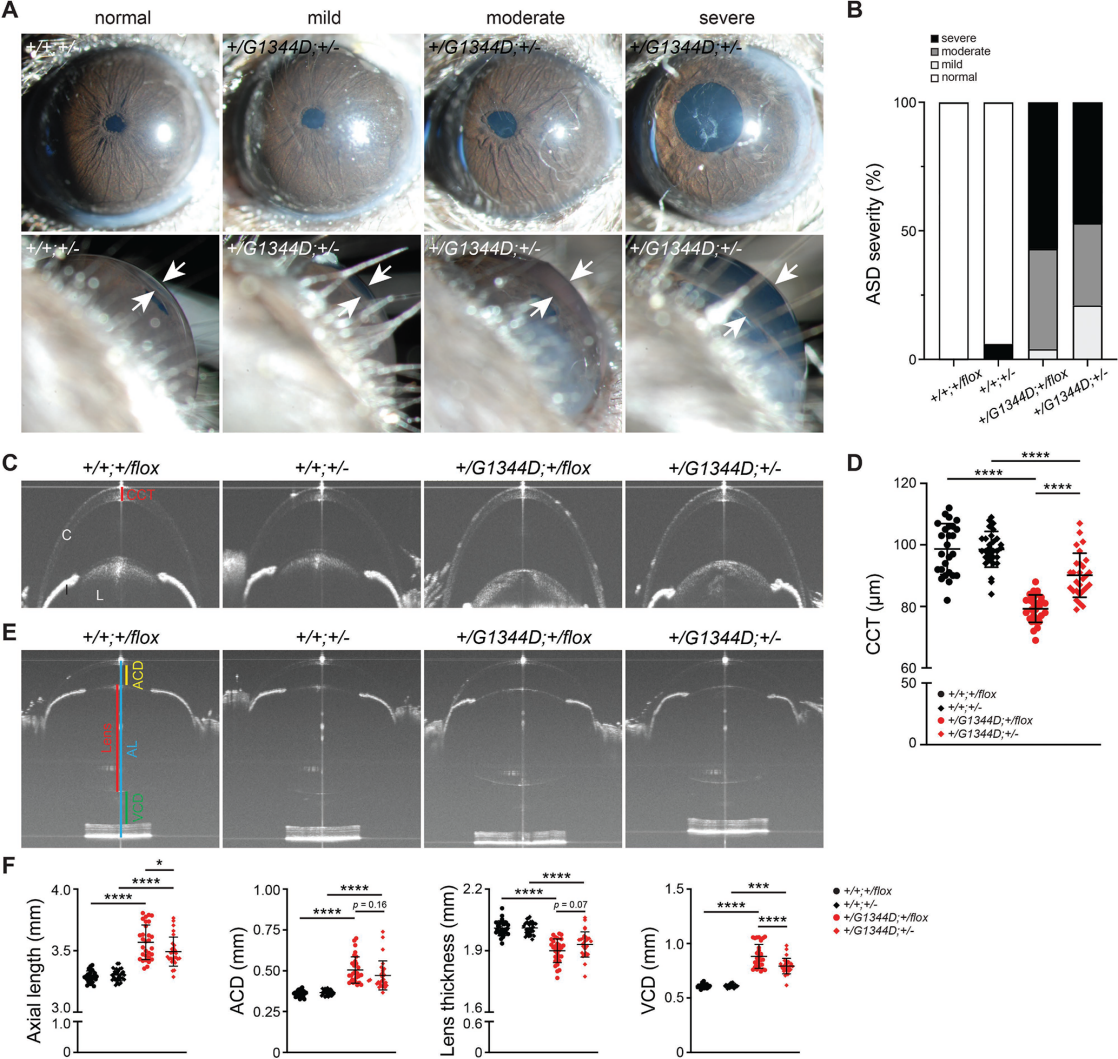
*Tgfbr2* heterozygosity reduces ASD in *Col4a1^+/G1344D^* mice. (**A**) Representative slit-lamp images of 1.3- to 1.5-month-old eyes showing examples of mild, moderate, and severe ASD, which typically manifested as dilated and tortuous iris vasculature, open pupil, cataracts, and enlarged anterior chamber. *Top panels* show frontal views of the eyes; *bottom panels* show side views of the eyes. *White arrows* indicate anterior chamber depth. (**B**) Histogram showing the percentage of eyes presenting with mild, moderate, and severe ASD in mice with the indicated genotype; *n* = 28 to 34 eyes for each genotype. (**C**, **D**) Representative OCT images of anterior segments (**C**) and quantification of CCT (**D**) showing significantly reduced CCT in 1.6- to 2.0-month-old *Col4a1^+/G1344D^* mice compared to control littermates that was ameliorated by *Tgfbr2* heterozygosity (comparing *Col4a1^+/G1344D^;Tgfbr2^+/^^−^* to *Col4a1^+/G1344D^;Tgfbr2^+/flox^* mice)*. Red bar* indicates CCT. C, cornea; I, iris; L, lens. (**E**, **F**) Representative OCT images of 1.6- to 2.0-month-old eyes (**E**) and quantification of various ocular biometric parameters (**F**) show increased ocular axial length (AL), anterior chamber depth (ACD), lens thickness, and vitreous chamber depth (VCD) in *Col4a1^+/G1344D^* mice compared to controls. *Tgfbr2* heterozygosity partially restored AL and VCD and tended to improve ACD and lens diameter in *Col4a1^+/G1344D^* mutant eyes. *Blue*, *yellow*, *red*, and *green bars* indicate ocular measurements for AL, ACD, lens thickness, and VCD, respectively; *n* = 26 to 30 eyes for each genotype. Data are presented as mean ± SD. **P < 0.01; ***P < 0.001; ****P < 0.0001, Fisher's exact test (**B**) and one-way ANOVA with Sidak's multiple comparison test (**D**, **F**).

To test the effect of *Tgfbr2* heterozygosity on ocular development, we first used slit-lamp biomicroscopy to examine the anterior segment (Figs. 2A, 2B). At 1.3 to 1.5 months of age, *Col4a1^+/G1344D^* mice have characteristic ASD, including enlarged pupils, cataract, iris pigment dispersion, tortuous and enlarged iris vasculature, and enlarged anterior chambers.^38^ ASD severity in *Col4a1* mutant mice is variable even for mice carrying the same mutation on a uniform genetic background.^25^^,^^33^^,^^38^ Out of 28 eyes from *Col4a1^+/G1344D^;Tgfbr2^+/flox^* mice, one eye (3.6%), 10 eyes (35.7%), and 17 eyes (60.7%) showed mild, moderate, or severe ASD, respectively. In contrast, six eyes (21.4%), nine eyes (32.1%), and 13 eyes (46.4%) out of 28 eyes from *Col4a1^+/G1344D^;Tgfbr2^+/^^−^* mice had mild, moderate, or severe ASD, respectively, suggesting that *Tgfbr2* heterozygosity might have a protective effect on ASD in *Col4a1^+/G1344D^* mice (Fig. 2B). We next performed a more quantitative analysis and measured the central corneal thickness (CCT).^33^ We found that *Col4a1^+/G1344D^* (*Col4a1^+/G1344D^;Tgfbr2^+/flox^* and *Col4a1^+/G1344D^;Tgfbr2^+/^^−^*) mice at 1.6 to 2.0 months of age had significantly reduced CCT compared to controls (*Col4a1^+/+^;Tgfbr2^+/flox^* and *Col4a1^+/+^;Tgfbr2^+/^^−^*), consistent with our previous observations (Figs. 2C, 2D).^33^ Importantly, CCT was significantly increased in *Col4a1^+/G1344D^;Tgfrbr2^+/–^* mice compared to *Col4a1^+/G1344D^;Tgfbr2^+/flox^* mice. *Col4a1^+/G1344D^* mice also have increased ocular axial length, anterior chamber depth, vitreous chamber depth, and decreased lens thickness.^33^ *Tgfbr2* heterozygosity improved these ocular biometric parameters in *Col4a1^+/G1344D^;Tgfbr2^+/–^* mice (Figs. 2E, 2F), although changes in anterior chamber depth and lens thickness did not reach statistical significance. Taken together, these results demonstrate that genetically reducing TGFBR2 levels has beneficial effects on ocular dysgenesis in *Col4a1^+/G1344D^* mice.

### *Tgfbr2* Heterozygosity Partially Rescues Anterior Synechiae and Appears to Ameliorate IOP in *Col4a1^+/G1344D^* Mice

**Figure 3.**
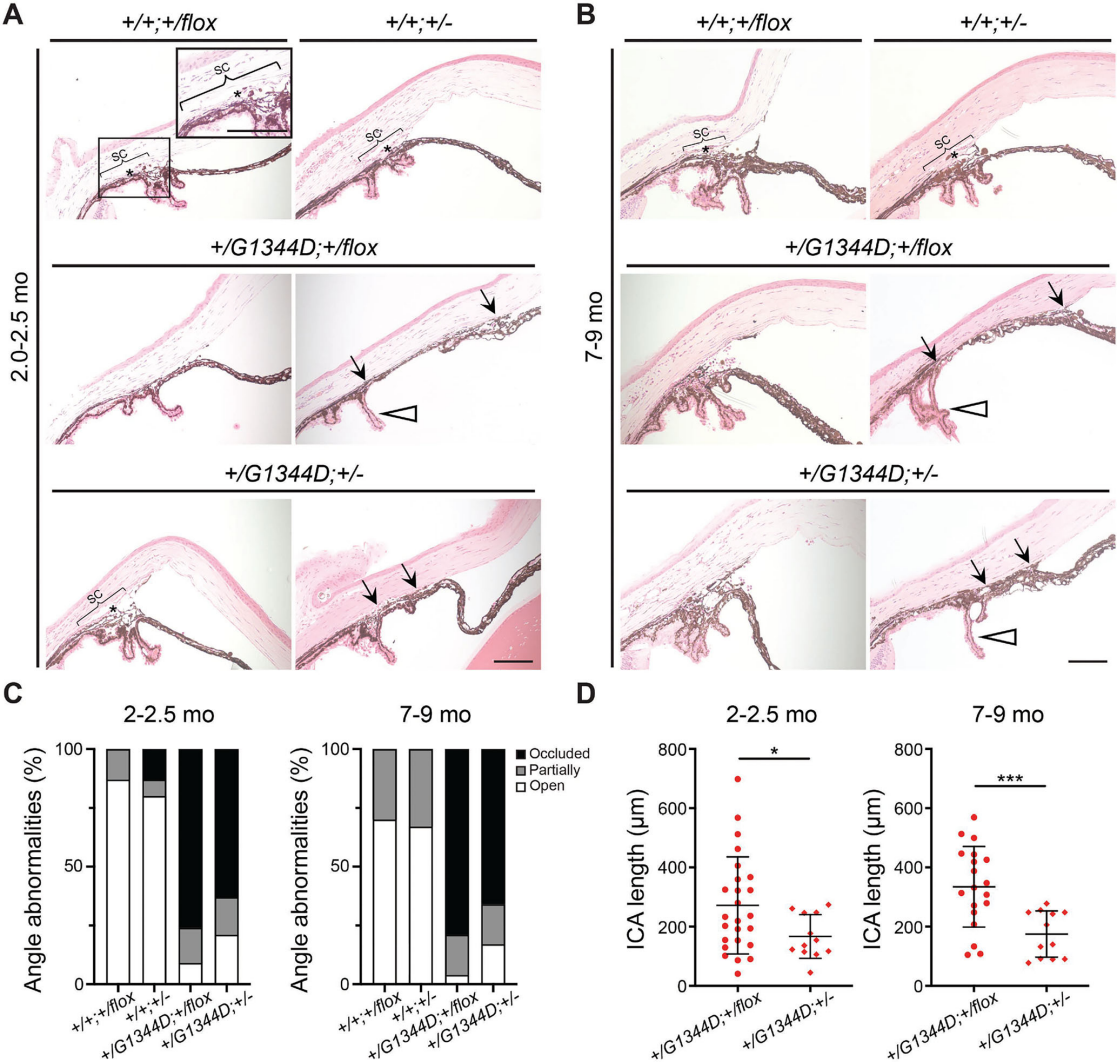
*Tgfbr2* heterozygosity ameliorates iridocorneal angle pathology in *Col4a1^+/G1344D^* mice. (**A**, **B**) Representative H&E-stained ocular sections from 2.0- to 2.5-month-old mice (**A**) and 7- to 9-month-old mice (**B**) showing that, in contrast to the presence of an open iridocorneal angle with identifiable TM (*asterisks*) and Schlemm's canal (SC; *bracket*) observed in *Col4a1^+/+^;Tgfbr2^+/flox^* and *Col4a1^+/+^;Tgfbr2^+/^^−^* eyes, *Col4a1* mutant eyes (*Col4a1^+/G1344D^;Tgfbr2^+/^*^flox^ and *Col4a1^+/G1344D^;Tgfbr2^+/^^−^*) showed variable degrees of pathology, including partially occluded (compressed TM and SC) or occluded (severe iridocorneal adhesion or ICA; *black arrows*). In addition, the ciliary body was often small and unfoliated (*open arrowheads*). *Scale bar*: 100 µm. (**C**) Frequency of open, partially occluded, or occluded angles with indicated genotype; *n* = 16 *Col4a1^+/+^;Tgfbr2^+/flox^*, *n* = 15 *Col4a1^+/+^;Tgfbr2^+/^^−^*, *n* = 33 *Col4a1^+/G1344D^;Tgfbr2^+/flox^*, and *n* = 19 *Col4a1^+/G1344D^;Tgfbr2^+/^^−^* angles at 2.0 to 2.5 months; *n* = 20 *Col4a1^+/+^;Tgfbr2^+/flox^*, *n* = 24 *Col4a1^+/+^;Tgfbr2^+/^^−^*, *n* = 24 *Col4a1^+/G1344D^;Tgfbr2^+/flox^*, and *n* = 18 *Col4a1^+/G1344D^;Tgfbr2^+/^^−^* angles at 7 to 9 months. (**D**) Quantification of ICA length showing that ICA worsens with ages in *Col4a1^+/G1344D^* eyes and can be partially prevented by *Tgfbr2* heterozygosity; *n* = 25 *Col4a1^+/G1344D^;Tgfbr2^+/flox^* and *n* = 12 *Col4a1^+/G1344D^;Tgfbr2^+/^^−^* angles at 2.0 to 2.5 months; *n* = 19 *Col4a1^+/G1344D^;Tgfbr2^+/flox^* and *n* = 12 *Col4a1^+/G1344D^;Tgfbr2^+/^^−^* angles at 7 to 9 months. Data are presented as mean ± SD. *P < 0.05; ***P < 0.001, Fisher's exact test (**C**) and Student's *t*-test with Welch's correction (**D**).

**Figure 4.**
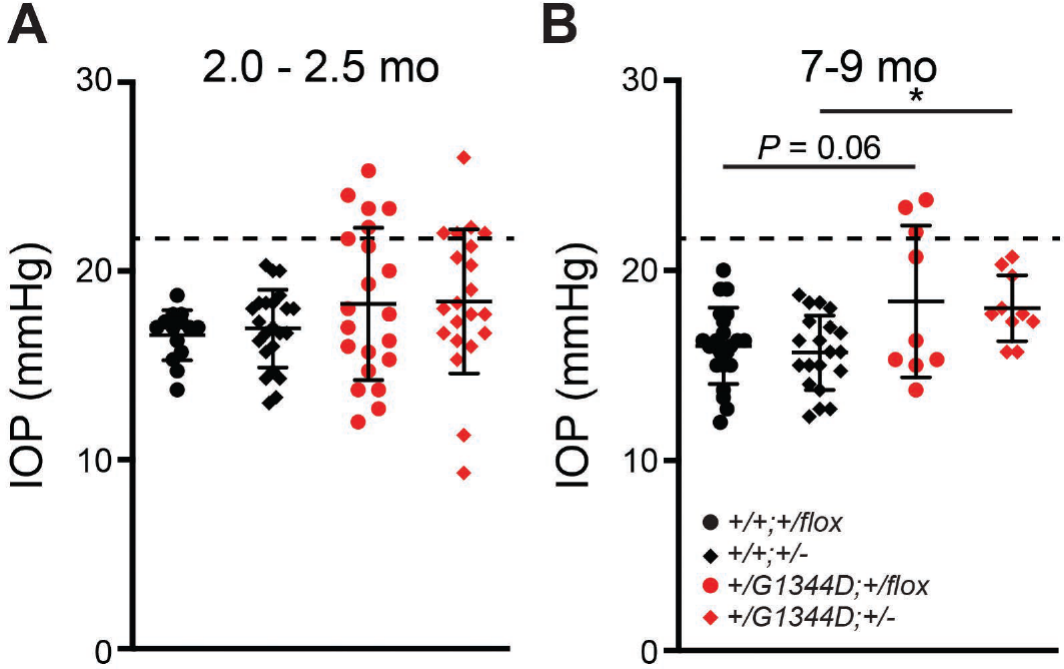
*Tgfbr2* heterozygosity affects IOP in *Col4a1^+/G1344D^* mice. (**A**, **B**) IOP measurements in mice at 2.0 to 2.5 months (**A**) and at 7 to 9 months (**B**). Multiple *Col4a1^+/G1344D^* eyes had high IOP (>21 mmHg) at both ages, and at 7 to 9 months the *Col4a1^+/G1344D^* eyes showed higher average IOPs than *Col4a1^+/+^* eyes. *Tgfbr2* heterozygosity at 7 to 9 months did not affect the mean of IOPs in *Col4a1^+/G1344D^* eyes, although the variation was smaller and the incidence of eyes with high IOP was reduced; *n* = 14 and 22 *Col4a1^+/+^;Tgfbr2^+/flox^* eyes, *n* = 23 and 20 *Col4a1^+/+^;Tgfbr2^+/^^−^* eyes, *n* = 21 and 9 *Col4a1^+/G1344D^;Tgfbr2^+/flox^* eyes, and *n* = 21 and 10 *Col4a1^+/G1344D^;Tgfbr2^+/^^−^* eyes at 2.0 to 2.5 months and at 7 to 9 months of age, respectively. *Dashed line* indicates IOP = 21 mmHg. Data are presented as mean ± SD. *P < 0.05, one-way ANOVA with Sidak's multiple comparison test.

Ocular drainage structures (trabecular meshwork [TM] and Schlemm's canal), located at the iridocorneal angle, control aqueous humor outflow and are crucial for IOP regulation.^45^ We previously showed that mice with a *Col4a1^Δ^^ex41^* mutation have iridocorneal adhesions and compressed or absent TM and Schlemm's canal.^46^ Moreover, *Col4a1^+/Δ^^ex41^* mice had abnormal IOP distributions, with approximately half having IOP higher than the highest controls.^46^ Because *Col4a1^+/G1344D^* mice also have deep anterior chambers, which can be an indication of high IOP, we performed histological analyses on *Col4a1^+/G1344D^* eyes to examine iridocorneal angle morphology (Fig. 3). We generated two groups of mice aged to 2.0 to 2.5 months old and 7 to 9 months old to investigate disease progression. At both ages, mice without the *Col4a1* mutation (*Col4a1^+/+^;Tgfbr2^+/flox^* and *Col4a1^+/+^;Tgfbr2^+/–^*) had a well-developed TM and Schlemm's canal, whereas the majority of the *Col4a1^+/G1344D^* mutant eyes (*Col4a1^+/G1344D^;Tgfbr2^+/flox^* and *Col4a1^+/G1344D^;Tgfbr2^+/–^*) had extensive anterior synechiae (peripheral iridocorneal adhesions) and ciliary body hypoplasia (Figs. 3A–3D). Notably, the extent of iridocorneal adhesions was reduced in *Col4a1^+/G1344D^;Tgfbr2^+/–^* eyes at both ages (Fig. 3D). To determine if iridocorneal adhesions led to increased IOP, we measured IOP with a rebound tonometer (Fig. 4). At around 2 months of age, some *Col4a1^+/G1344D^* mice begin developing corneal opacities or scarring that worsens with age and can affect the accuracy of IOP measurement^47^; therefore, eyes with visible corneal defects were excluded, including five eyes (19.2%) and 10 eyes (45.5%) from the 2.0- to 2.5-month-old and the 7- to 9-month-old *Col4a1^+/G1344D^;Tgfbr2^+/flox^* mice, respectively, and one eye (4.5%) and seven eyes (38.9%) eyes from the 2.0- to 2.5-month-old and 7- to 9-month-old *Col4a1^+/G1344D^;Tgfbr2^+/^^−^* mice, respectively. The percentage of eyes excluded was smaller in *Col4a1^+/G1344D^;Tgfbr2^+/flox^* mice*,* consistent with our previous observation showing a protective role for *Tgfbr2* heterozygosity in ASD of *Col4a1^+/G1344D^* mice. The remaining *Col4a1^+/G1344D^* eyes showed variable IOP, including eyes with IOP > 21 mmHg in both age groups. At 7 to 9 months of age, the *Col4a1^+/G1344D^* eyes had higher average IOPs compared to *Col4a1^+/+^* eyes. IOPs from *Col4a1^+/G1344D^;Tgfbr2^+/flox^* mice appeared to have a bimodal profile, and approximately a third exceeded 21 mmHg. Although *Tgfbr2* heterozygosity did not affect average IOP in *Col4a1^+/+^* eyes, it reduced IOP variability, and none of the *Col4a1^+/G1344D^;Tgfbr2^+/–^* mice had high IOPs over 21 mmHg.

### *Tgfbr2* Heterozygosity Partially Rescues Retinal and Optic Nerve Defects in *Col4a1^+/G1344D^* Mice

**Figure 5.**
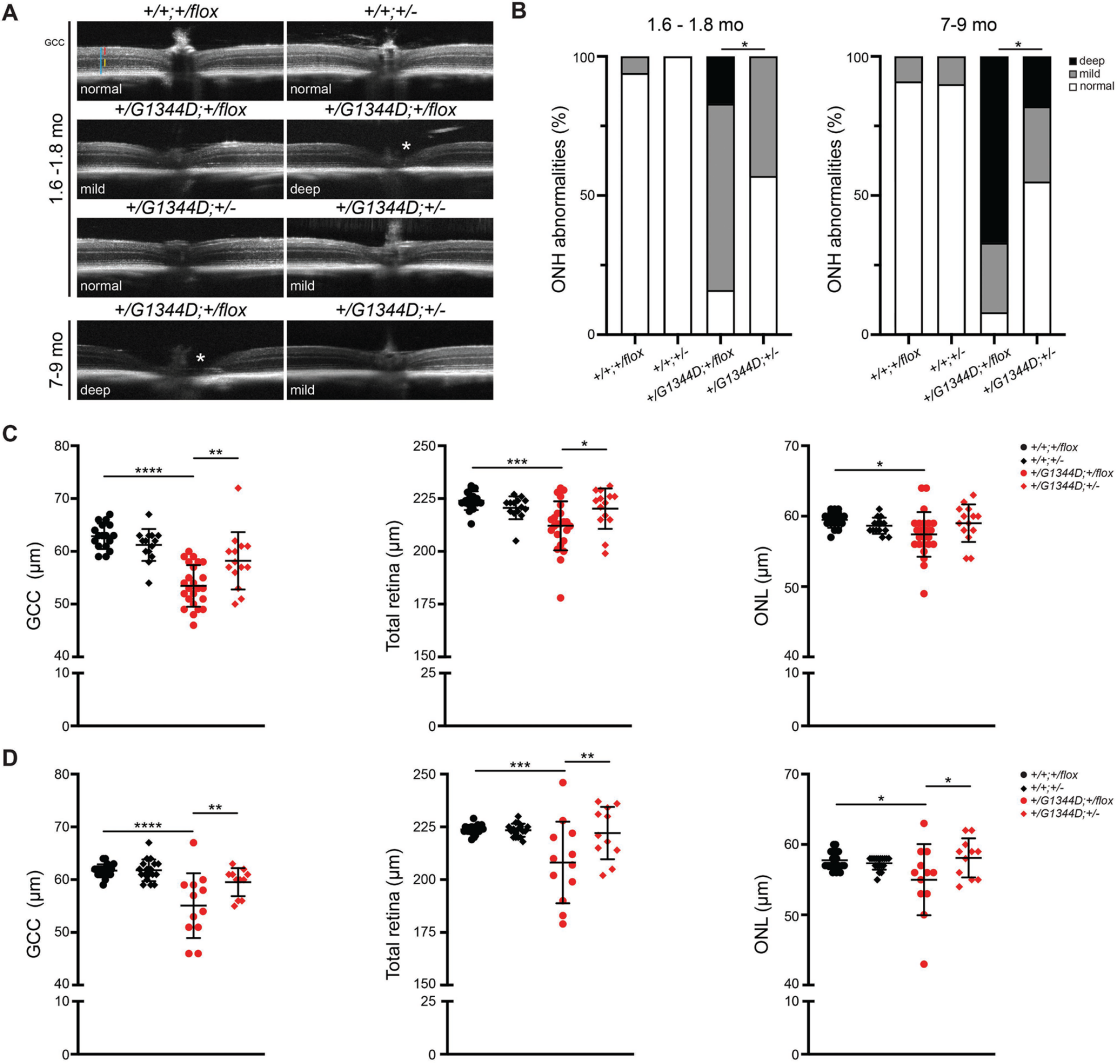
*Tgfbr2* heterozygosity reduced the frequency of optic nerve head excavation and improved retinal thickness in *Col4a1^+/G1344D^* mice. (**A**) Representative OCT images showing the central retina and optic nerve head in mice at 1.6 to 1.8 months and at 7 to 9 months. Although the optic nerve head appeared to be flat in wild-type eyes, *Col4a1^+/G1344D^* eyes showed optic nerve head excavation (*asterisks*) with variable severity. *Blue*, *red*, and *yellow bars* indicate ocular measurements for total retinal, GCC, and ONL thickness, respectively. A magnified image is also shown in Supplementary Figure S2. (**B**) Frequency of normal, mildly, or deeply excavated optic nerve heads in wild-type or *Col4a1^+/G1344D^* mice with or without *Tgfbr2* heterozygosity at 1.6 to 1.8 months or 7 to 9 months of age. The incidence of severe cupping increased with age in *Col4a1^+/G1344D^* eyes, and it was reduced with *Tgfbr2* heterozygosity. ONH, optic nerve head. (**C**, **D**) Quantification of thickness of different retinal layers by OCT biometry. In both age groups, *Col4a1^+/G1344D^* eyes had thinner GCC layers, reduced total retinal thickness, and thinner outer nuclear layer (ONL). When *Tgfbr2* was inactivated, the thickness of those layers was partially restored (except the ONL in the young age group); *n* = 17 and 21 *Col4a1^+/+^;Tgfbr2^+/flox^* eyes, *n* = 14 and 18 *Col4a1^+/+^;Tgfbr2^+/^^−^* eyes, *n* = 24 and 12 *Col4a1^+/G1344D^;Tgfbr2^+/flox^* eyes, and *n* = 14 and 11 *Col4a1^+/G1344D^;Tgfbr2^+/^^−^* eyes at 1.6 to 1.8 months and 7 to 9 months of age, respectively. Data are presented as mean ± SD. *P < 0.05; **P < 0.1; ***P < 0.001; ****P < 0.0001, Fisher's exact test (**B**) and one-way ANOVA with Sidak's multiple comparison test (**C**, **D**).

To assess the impact of the *Col4a1^+/G1344D^* mutation and TGFβ signaling on the retina and optic nerve head, we first performed in vivo OCT analyses (Fig. 5, Supplementary Fig. S2). Although all retinas were visible at a young age, corneal or lens opacities in some eyes from mutant mice at 7 to 9 months prevented imaging, and these mice (45.5% and 38.9% for *Col4a1^+/G1344D^;Tgfbr2^+/flox^* and *Col4a1^+/G1344D^;Tgfbr2^+/^^−^*, respectively) were excluded from the following analyses. In contrast to control retinas, *Col4a1^+/G1344D^* eyes often showed cupped optic nerve heads that were occasionally V-shaped, consistent with tissue loss or optic nerve head excavation (Fig. 5A), and the prevalence of abnormal optic nerve heads increased with age (Fig. 5B). In the context of *Tgfbr2* heterozygosity, the percentage of eyes with abnormal optic nerve heads was reduced in *Col4a1^+/G1344D^* mice at both ages (Fig. 5B). To quantify the impact of *Tgfbr2* reduction on the inner retina, we measured the thickness of the GCC, which is the sum of thicknesses of the retinal NFL, retinal ganglion cell layer, and inner plexiform layer, which are potentially involved in glaucomatous damage and have been used as a biomarker to monitor optic neuropathy in mouse models and in humans.^48^ *Col4a1^+/G1344D^* mice had a thinner GCC compared to controls, and the GCC thickness in *Col4a1^+/G1344D^;Tgfbr2^+/–^* mice was significantly improved in both age groups (Figs. 5C, 5D). The total retinal thickness was also reduced in *Col4a1^+/G1344D^* mice, which was also attributable to reduced thickness of the outer nuclear layer (ONL). This was consistent with previous reports showing that the outer retinal defects could occur in humans or rodents with angle closure glaucoma and prolonged IOP elevation.^49^^,^^50^ More importantly, *Tgfbr2* heterozygosity significantly improved both ONL and total retinal thickness.

**Figure 6.**
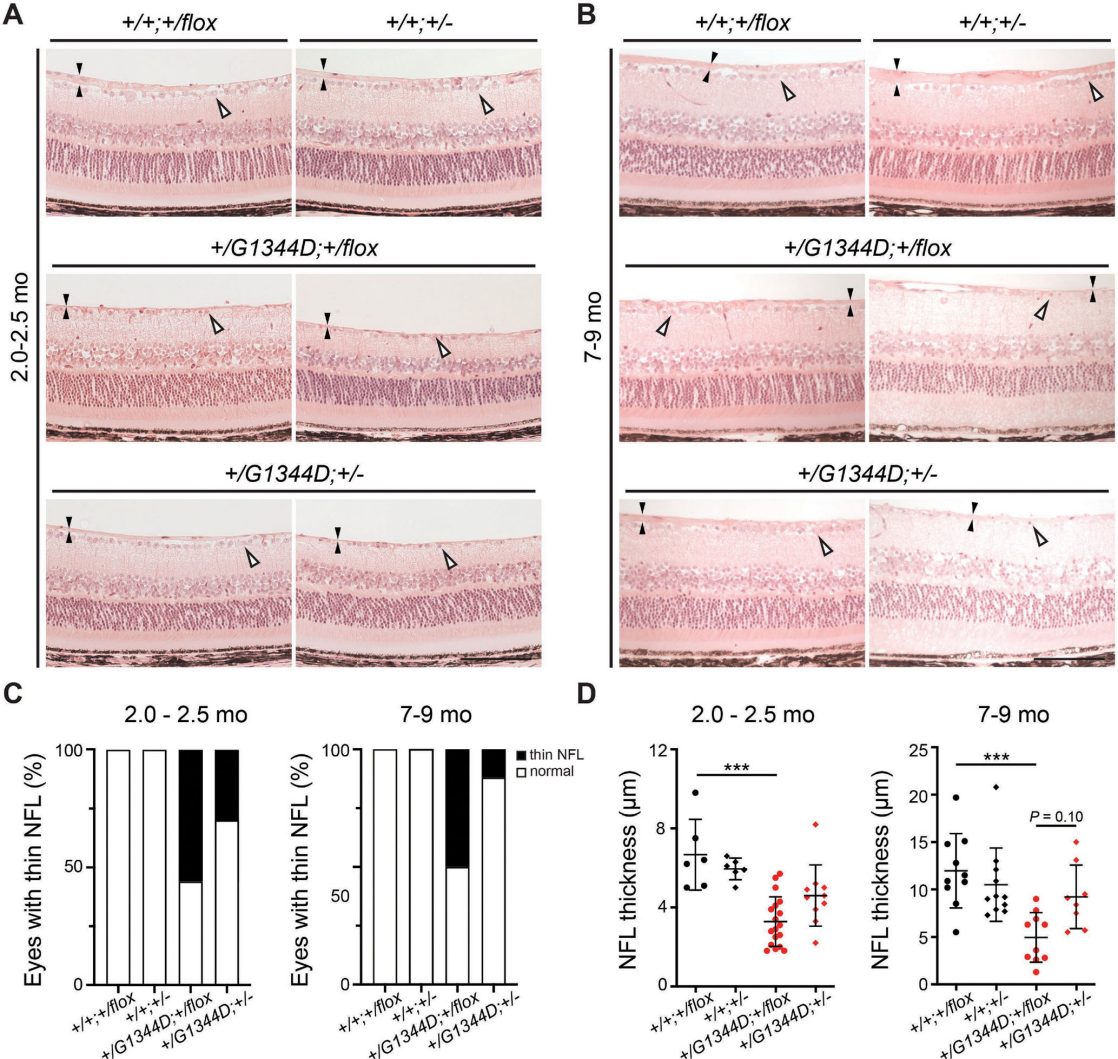
Histological analyses of the retina revealed a protective role of *Tgfbr2* heterozygosity in *Col4a1^+/G1344D^* mice. (**A**, **B**) Representative histological images of the central retina in *Col4a1^+/+^* or *Col4a1^+/G1344D^* mice with or without *Tgfbr2* heterozygosity at 2.0 to 2.5 months of age (**A**) or 7 to 9 months of age (**B**). *Black arrowheads* indicate the NFLs, and *open arrowheads* indicate cell bodies in the RGC layer. Although some *Col4a1^+/G1344D^* eyes appeared to be normal with a robust NFL and a continuous layer of cells, others showed thin NFLs accompanied with cell loss. *Scale bar*: 100 µm. (**C**) Frequency of eyes with thin NFLs and cell loss in mice with indicated genotype and age. Although all control retinas were healthy, approximately 50% of the *Col4a1^+/G1344D^;Tgfbr2^+/flox^* eyes had thin NFLs and discontinued retinal ganglion cells. *Tgfbr2* heterozygosity appeared to reduce the frequency of eyes with thin NFLs and cell loss in both age groups. (**D**) Quantification of the NFL thickness. We observed a trend toward protection in mice with *Tgfbr2* heterozygosity at 7 to 9 months of age; *n* = 6 and 10 *Col4a1^+/+^;Tgfbr2^+/flox^* eyes, *n* = 6 and 11 *Col4a1^+/+^;Tgfbr2^+/^^−^* eyes, *n* = 18 and 10 *Col4a1^+/G1344D^;Tgfbr2^+/flox^* eyes, and *n* = 10 and 8 *Col4a1^+/G1344D^;Tgfbr2^+/^^−^* eyes at 2.0 to 2.5 months and 7 to 9 months of age, respectively. ***P < 0.001, Fisher's exact test (**C**) and Kruskal–Wallis test with Dunn's multiple comparison test (**D**).

**Figure 7.**
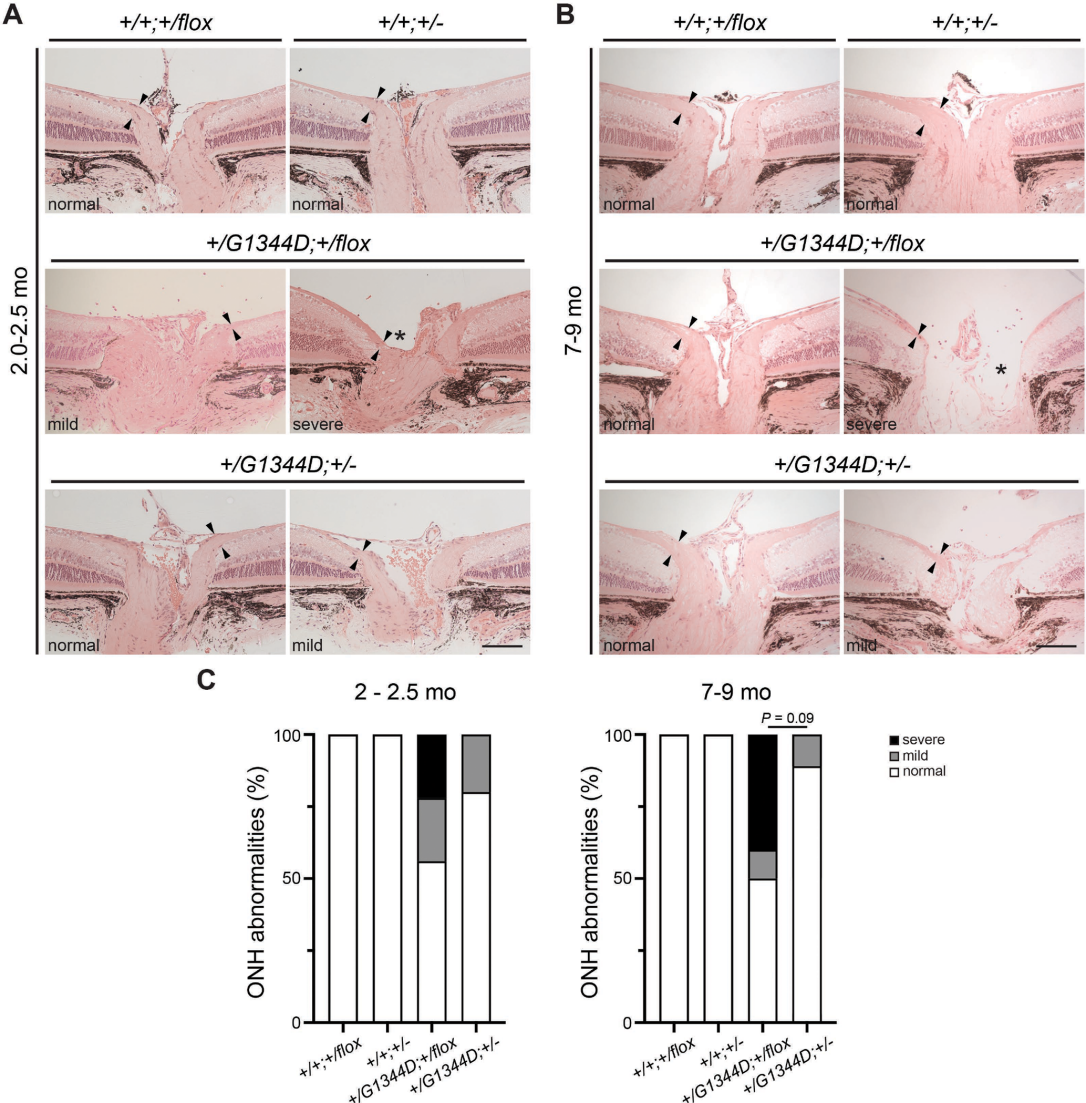
*Tgfbr2* heterozygosity prevents optic nerve head damage in *Col4a1^+/G1344D^* mice. (**A**, **B**) Representative images of H&E-stained ocular sections from mice at 2.0 to 2.5 months (**A**) and at 7 to 9 months (**B**). Although some *Col4a1^+/G1344D^* eyes had a robust NFL and normal optic nerve head morphology, the others showed a thin NFL and tissue loss in the optic nerve head. *Black arrowheads* indicate the NFL, and the *asterisk* indicates optic nerve head excavation. *Scale bar*: 100 µm. (**C**) Frequency of normal, mildly, or deeply excavated optic nerve heads in mice with indicated genotype and age. *Tgfbr2* heterozygosity appeared to reduce the frequency of eyes with optic nerve head excavation at both ages examined, and a trend toward significance was observed at 7 to 9 months of age. ONH, optic nerve head; *n* = 6 and 10 *Col4a1^+/+^;Tgfbr2^+/flox^* eyes, *n* = 6 and 11 *Col4a1^+/+^;Tgfbr2^+/^^−^* eyes, *n* = 18 and 10 *Col4a1^+/G1344D^;Tgfbr2^+/flox^* eyes, and *n* = 10 and 8 *Col4a1^+/G1344D^;Tgfbr2^+/^^−^* eyes at 2.0 to 2.5 months and 7 to 9 months of age, respectively. Fisher's exact test for **C**.

**Figure 8.**
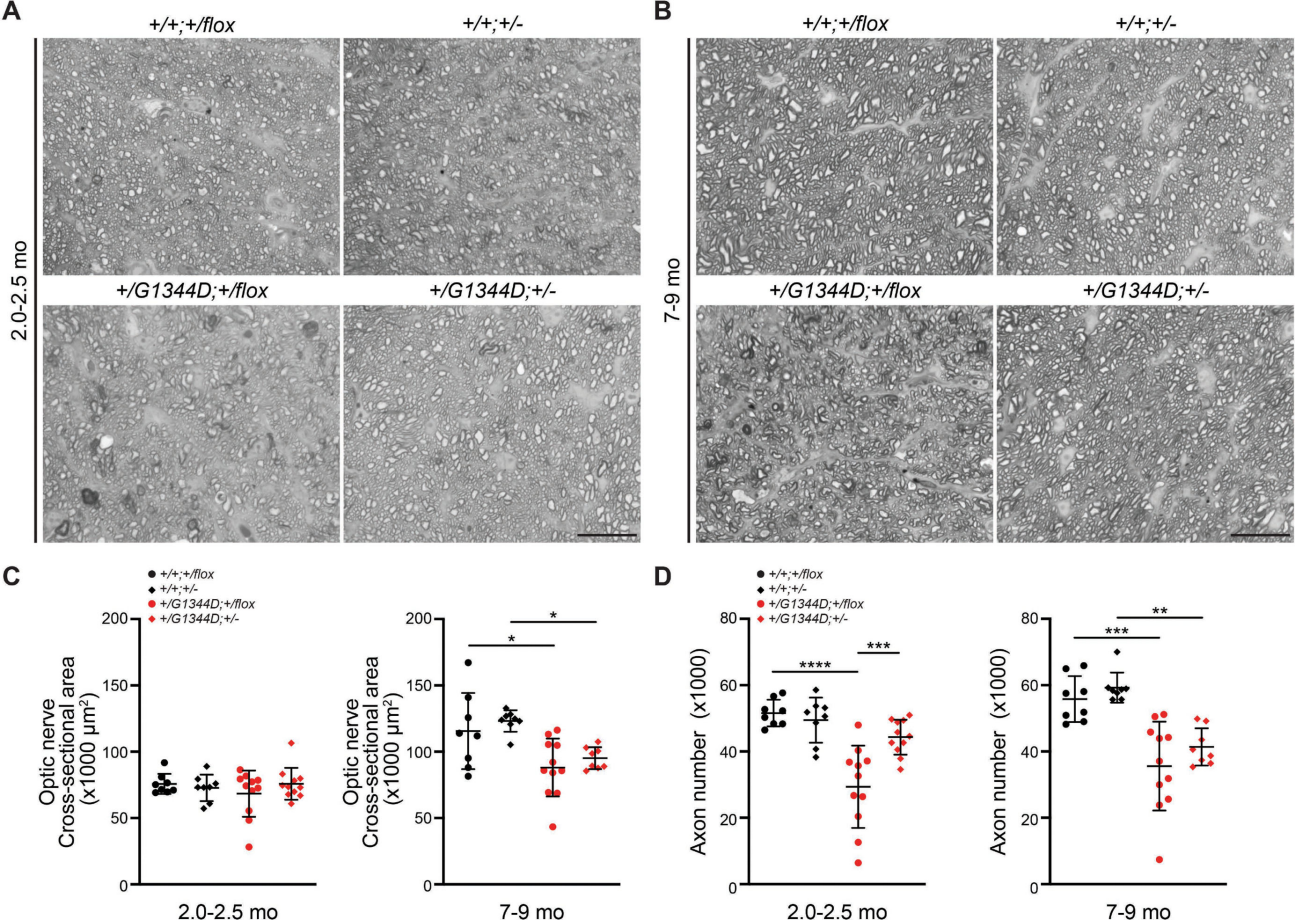
*Tgfbr2* heterozygosity partially prevents axonal degeneration in optic nerves from *Col4a1^+/G1344D^* mice. (**A**, **B**) Representative cross-sections of PPD-stained optic nerves from mice at 2.0 to 2.5 months (**A**) or 7 to 9 months (**B**). (**C**, **D**) Quantification of cross-sectional area (**C**) and total axon number (**D**) showing that, in contrast to the healthy myelinated axons observed in wild-type optic nerves, *Col4a1^+/G1344D^* nerves contained numerous degenerated axons, as indicated by darkly stained axoplasm, and that *Tgfbr2* heterozygosity partially prevented axonal loss in *Col4a1^+/G1344D^* optic nerves; *n* = 8 and 8 *Col4a1^+/+^;Tgfbr2^+/flox^* eyes, *n* = 8 and 8 *Col4a1^+/+^;Tgfbr2^+/^^−^* eyes, *n* = 11 and 11 *Col4a1^+/G1344D^;Tgfbr2^+/flox^* eyes, and *n* = 11 and 8 *Col4a1^+/G1344D^;Tgfbr2^+/^^−^* eyes at 2.0 to 2.5 months and at 7 to 9 months of age, respectively. *Scale bar*: 20 µm. Data are presented as mean ± SD. *P < 0.05; **P < 0.1; ***P < 0.001; ****P < 0.0001, one-way ANOVA with Sidak's multiple comparison test.

To validate these findings, we next carried out histological analyses on retinal sections of eyes that underwent OCT. Consistent with OCT findings, we found that a proportion of *Col4a1^+/G1344D^* eyes had severely reduced NFLs and fewer cells in the retinal ganglion cell layer in both age groups (Fig. 6) and that the frequency appeared to be lower in *Col4a1^+/G1344D^* mice with *Tgfbr2* heterozygosity (Fig. 6C). When we quantified NFL thickness, we found a significant reduction in *Col4a1^+/G1344D^;Tgfbr2^+/flox^* mice at both ages and that *Tgfbr2* heterozygosity had a trend toward protection at 7 to 9 months of age (Fig. 6D). Moreover, at the level of the optic nerve head, a proportion of *Col4a1^+/G1344D^* eyes had thinner NFL and optic nerve head excavation, which progressively worsened with age (Fig. 7). Importantly, *Tgfbr2* heterozygosity showed a trend toward protection in *Col4a1^+/G1344D^* mice at 7 to 9 month of age. We next sought to determine if *Col4a1^+/G1344D^* mice have retinal ganglion cell axon damage in the optic nerve consistent with glaucoma. We sectioned optic nerves and stained them with PPD, which differentially stains the myelin sheaths of healthy axons versus the axoplasm of damaged or dying axons. Although most control optic nerves appeared to be normal, optic nerves from *Col4a1^+/G1344D^* mice at both ages showed signs of axon degeneration as indicated by darkly stained or demyelinated axons (Figs. 8A, 8B). We quantified the cross-sectional areas of the optic nerves (Fig. 8C) and observed significant reduction in *Col4a1^+/G1344D^* mice at 7 to 9 months of age but not at 2.0 to 2.5 months of age. Moreover, quantification of healthy, myelinated axons revealed a significant reduction in axon number in *Col4a1^+/G1344D^* mice at both ages (Fig. 8D). Although *Tgfbr2* heterozygosity significantly increased the number of axons in *Col4a1^+/G1344D^* mice at 2 to 2.5 months, it did not reach statistical significance at 7 to 9 months; however, fewer samples had extremely low axon counts. Taken together, our data demonstrate that *Col4a1^+/G1344D^* mutant mice exhibit hallmarks of glaucomatous neurodegeneration including thinning of the NFL, retinal ganglion cell loss, optic nerve head excavation, and optic nerve axon loss, which can be partially prevented by *Tgfbr2* heterozygosity.

## Discussion

Type IV collagens are major BM constituents that serve as multifunctional cell adhesion and signaling platforms. *COL4A1* and *COL4A2* mutations cause Gould syndrome, a multisystem disorder mainly associated with cerebrovascular, ocular, renal, and muscular defects with variable disease onset, penetrance, and severity.^15^^,^^16^ The biological functions of the collagen α1α1α2(IV) network remain largely unknown, and the mechanisms underlying Gould syndrome pathogenesis are unclear. Our previous studies showed that *Col4a1* mutant mice have elevated TGFβ signaling and that reducing TGFβ signaling improves anterior segment and cerebrovascular defects.^33^^–^^35^ Using mice that have reduced levels of TGFBR2, a key receptor of all three TGFβ ligand isoforms, here we examined the role of TGFβ signaling in the context of hallmarks of glaucoma including IOP, optic nerve, and retinal NFL parameters. We found that *Col4a1^+/G1344D^* mice have multiple pathological hallmarks of glaucoma and that *Tgfbr2* heterozygosity partially prevents these phenotypes in *Col4a1^+/G1344D^* mice.

The majority of the ocular anterior segment is derived from the periocular mesenchyme.^51^ Precise regulation of TGFβ signaling activity is critical for differentiation of the periocular mesenchyme, as absence of TGFβ2 or TGFBR2 or TGFβ1 overexpression lead to malformation of anterior segment structures.^52^^–^^54^ Consistent with its role in the developing anterior segment, *Tgfbr2* heterozygosity appeared to decrease ASD severity and increase corneal thickness in *Col4a1^+/G1344D^* mice. This observation is consistent with our previous findings showing that reducing TGFβ ligands protects against ASD.^33^ In addition, here we show that *Col4a1^+/G1344D^* eyes also have anterior synechiae by 2 months of age. Although we were unable to assess the extent of circumferential synechiae occlusion, we observed a reduction in the length of adhesion in *Col4a1^+/G1344D^* eyes with *Tgfbr2* heterozygosity, suggesting that altered TGFβ signaling might also contribute to this phenotype. The iridocorneal angle in mice is still developing postnatally,^2^ and it is possible that the angle abnormalities are due to failed morphogenesis of the ocular drainage structures and separation of iris from cornea. Alternatively, ectopic expression of TGFβ1 could induce a fibrotic response and lead to pathological changes, including anterior synechiae.^55^^,^^56^ Regardless, given the essential roles of TGFβ signaling in anterior segment development and fibrosis, it is conceivable that *Tgfbr2* heterozygosity could have protective effects in *Col4a1* mutant mice by influencing either or both of these processes.

High IOP is an important consequence of anterior synechiae as it blocks aqueous humor outflow from ocular drainage structures. Here, we showed that IOPs in *Col4a1^+/G1344D^* eyes tend to be higher than in controls. Although *Tgfbr2* heterozygosity in *Col4a1^+/G1344D^* mice might reduce the extent of anterior synechiae, it did not seem to lower IOP on average, although the IOP variation and the frequency of eyes with high IOP were reduced. Because corneal abnormalities interfere with tonometer IOP measurement accuracy, approximately 40% of *Col4a1^+/G1344D^* eyes were excluded, making our study underpowered and potentially masking a protective effect on IOP. Consistent with a protective effect of *Tgfbr2* heterozygosity, fewer eyes having corneal opacity were excluded in the *Col4a1^+/G1344D^;Tgfbr2^+/–^* group than in the *Col4a1^+/G1344D^;Tgfbr2^+/flox^* group. OCT imaging and histological analyses revealed that *Col4a1^+/G1344D^* eyes have characteristic features of glaucoma, including thinning of the NFL, loss of retinal ganglion cells, thinner GCC, optic nerve head excavation, and optic neuropathy, and *Tgfbr2* heterozygosity reduced the frequency of eyes showing glaucomatous damage. TGFβ signaling has been widely implicated in glaucoma. TGFβ2 levels are elevated in the aqueous humor of patients with primary open-angle glaucoma.^57^^,^^58^ Activation of the TGFβ pathway can promote ECM synthesis and crosslinking, modulate the cytoskeleton of TM cells, and increase cell contractility, thus increasing aqueous humor outflow resistance and IOP.^59^^–^^61^ Elevated IOP might also induce mechanical stress to the optic nerve head, leading to impaired axonal transport and neurotrophic deprivation, which augment optic nerve axon degeneration.^62^ The protective effect of reduced TGFβ signaling on glaucomatous pathology could also occur in an IOP- independent manner. Significant amounts of TGFβ2 were found at the optic nerve head of glaucoma patients, and TGFβ2 can directly increase ECM production by cells isolated from the optic nerve head.^63^^–^^65^ Reactive astrocytes are thought to be the major source of TGFβ2^65^^,^^66^; therefore, it is possible that *Tgfbr2* heterozygosity might be protective by modulating astrocyte activity that is critical for retinal ganglion cell health.

Because TGFBR2 is the only type II receptor for all three TGFβ ligands, it is conceivable that reducing TGFBR2 levels have greater effect than reducing one ligand isoform in tissues that express all three ligands. In normal eyes, TGFβ2 is the predominant form and is highly expressed in the lens, whereas TGFβ1 and TGFβ3 show little expression.^52^^,^^67^ In *Col4a1* mutant eyes, the spatial and temporal expression of TGFβ ligands is not clear. We previously showed that TGFβ1 and TGFβ2 have distinct roles in ocular pathology in *Col4a1* mutant mice.^33^ Although TGFβ2 reduction in *Col4a1* mutant mice only rescues CCT, reducing TGFβ1 normalized anterior chamber depth, lens diameter, and vitreous chamber depth in *Col4a1* mutant mice but not CCT.^33^ Here, we found that reducing TGFBR2 levels ameliorated all aforementioned parameters, supporting the suggestion that both TGFβ1 and TGFβ2 mediate ocular pathology in *Col4a1* mutant mice. How *Col4a1* mutations lead to upregulated TGFβ signaling is still not known. Type IV collagens can bind directly to TGFβ family members,^18^^,^^68^^,^^69^ and it is possible that extracellular collagen α1α1α2(IV) deficiency increases TGFβ bioavailability. Alternatively, type IV collagens have multiple binding sites for integrin α1β1 that can suppress TGFBR2 activity.^70^^–^^72^ Thus, it is possible that collagen α1α1α2(IV) deficiency results in de-repression of TGFBR2 activity via integrin-mediated mechanisms. Whether elevated TGFβ signaling is caused by ectopic expression or activation of TGFβ ligands, by their improved bioavailability, or by altered downstream signaling mediators and modulators remains to be investigated.

In conclusion, we provide evidence suggesting that elevated TGFβ signaling is a pathogenic mechanism contributing to glaucomatous features observed in *Col4a1* mutant mice. Elevated TGFβ signaling may participate in various manifestations associated with Gould syndrome, highlighting it as a potential therapeutic target.

## Supplementary Material

Supplement 1

Supplement 2
