# Supplementary material for: TGFβ Signaling Dysregulation May Contribute to COL4A1-Related Glaucomatous Optic Nerve Damage
Source: Invest Ophthalmol Vis Sci. 2024 May 8;65(5):15. doi: 10.1167/iovs.65.5.15 (PMC11090142; doi:10.1167/iovs.65.5.15)
Supplement: Supplement 1 [file iovs-65-5-15_s001.pdf]

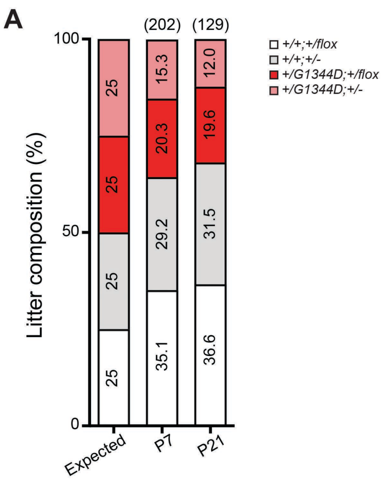

Fig. S1. Litter composition of *Col4a1*$^{+/G1344D}$ mice with or without *Tgfbr2* heterozygosity. (A) Consistent with previous reports, *Col4a1*$^{+/G1344D}$ mice show reduced survival at P7 and weaning age (P21) compared to wildtype mice. n = 202 and 129 mice at P7 or P21, respectively.

A

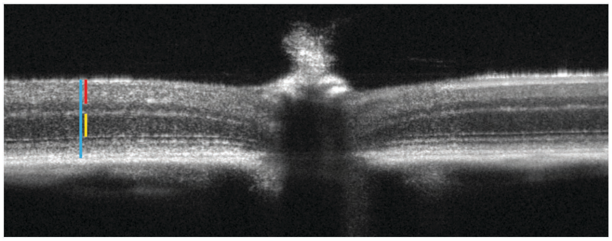

Fig S2. Examples of OCT measurements for different retinal layers. Blue, red, and yellow bars indicate ocular measurements for total retinal, GCC, and ONL thickness, respectively. The same image was also shown in Fig. 5A.
