# Supplementary material for: TGFβ Signaling Dysregulation May Contribute to COL4A1-Related Glaucomatous Optic Nerve Damage
Source: Invest Ophthalmol Vis Sci. 2024 May 8;65(5):15. doi: 10.1167/iovs.65.5.15 (PMC11090142; doi:10.1167/iovs.65.5.15)
Supplement: Supplement 2 [file iovs-65-5-15_s002.pdf]

Table S1. List of primers.

Gene	**Forward Primer (5' to 3')**	**Reverse Primer (5' to 3')**
Gapdh	AGGTCGGTGTGAACGGATTTG	TGTAGACCATGTAGTTGAGGTCA
Hprt1	TGACACTGGCAAAACAATGCA	GGTCCTTTTCACCAGCAAGCT
Serpine1	GGCATGAGCTGTGCCCTTCT	CAGATGACCACAGCGGGGAA
Tgfbr2(F1&R1)	CCGCTGCATATCGTCCTGTG	CACTTGGATAATGACCAACAAC
Tgfbr2(F2&R2)	CACTTGCGACAACCAGAA	CAAACCGTCTCCAGAGTAATG
